# Supplementary figures and images for: Non-allergic eye rubbing is a major behavioral risk factor for keratoconus
Source: PLoS One. 2023 Apr 13;18(4):e0284454. doi: 10.1371/journal.pone.0284454 (PMC10101517; doi:10.1371/journal.pone.0284454)

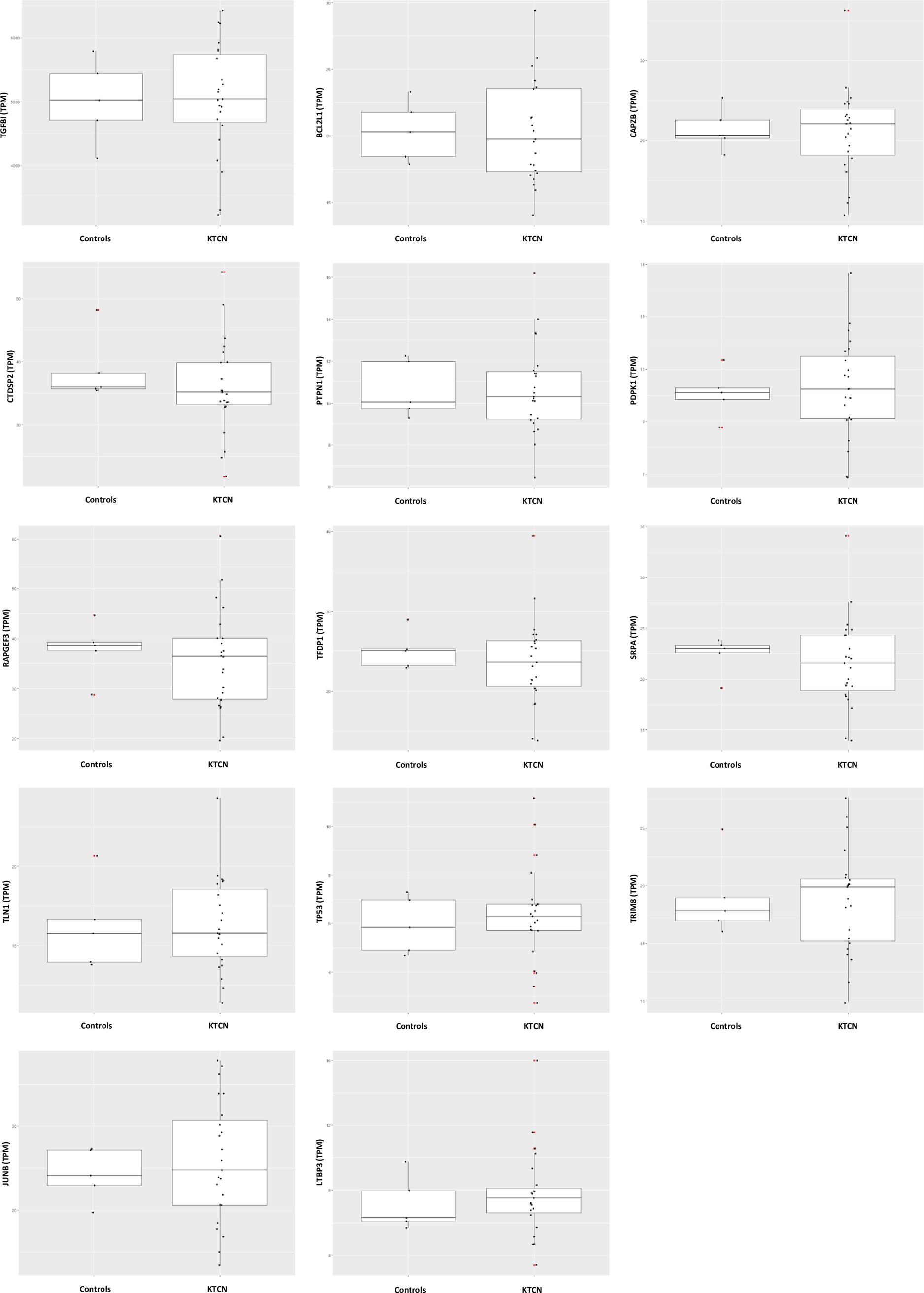

Supplement: S1 Fig — The boxplots presenting the expression of selected genes, TGFBI, BCL2L1, CAPZB, CTDSP2, PTPN1, PDPK1, RAPGEF3, TFDP1, SRPRA, TLN1, TP53, TRIM8, JUNB, and LTBP3 (in TPM, Transcripts per Million), in the cone (central) region of corneal epithelium in the studied groups of patients with KTCN and controls. (TIF) [file pone.0284454.s001.tif]

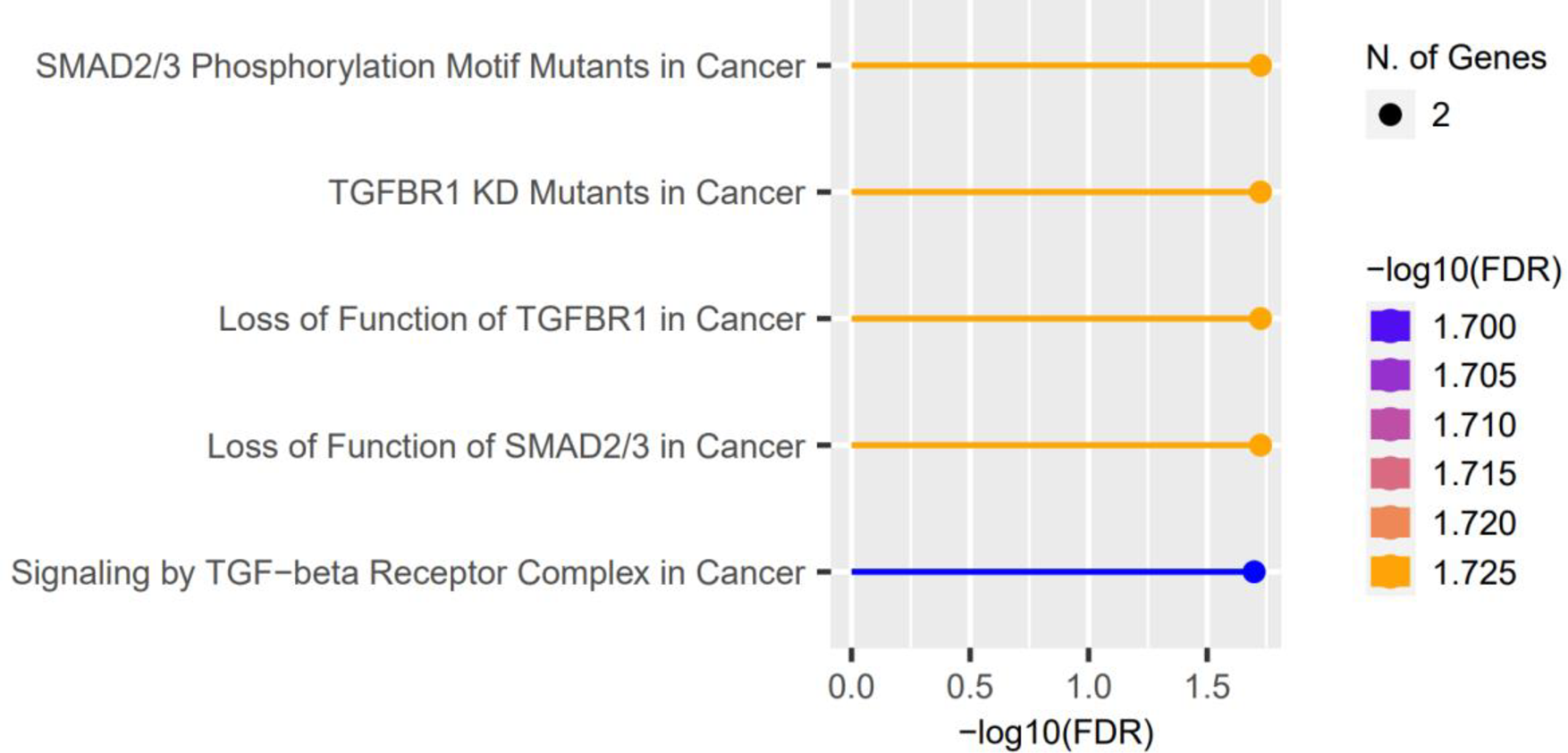

Supplement: S2 Fig — Results of pathway enrichment analysis for genes whose expression in corneal epithelium was found to be correlated with the allergy status. Transcriptome data was obtained for males and females from both study groups. The Reactome database was chosen as the source of the pathways. (TIF) [file pone.0284454.s002.tif]
