# Supplementary material for: Non-allergic eye rubbing is a major behavioral risk factor for keratoconus
Source: PLoS One. 2023 Apr 13;18(4):e0284454. doi: 10.1371/journal.pone.0284454 (PMC10101517; doi:10.1371/journal.pone.0284454)
Supplement: S1 Table — (DOCX) [file pone.0284454.s003.docx]

**S1 Table. Results of clinical comparison of male and female patients with KTCN**

|  | **Males with KTCN (n=97)** | | **Females with KTCN (n=21)** | |
| --- | --- | --- | --- | --- |
|  | **x** $\boldsymbol{\pm}$ **SD** | **Median** | **x**$\boldsymbol{\pm}$ **SD** | **Median** |
| UDVA OD | 0.49 ± 0.38 | 0.40 | 0.32 ± 0.28 | 0.20 |
| BDVA OD | 0.81 ± 0.35 | 0.90 | 0.73 ± 0.34 | 0.85 |
| UDVA OS | 0.43 ± 0.34 | 0.30 | 0.52 ± 0.37 | 0.40 |
| BDVA OS | 0.77 ± 0.35 | 0.90 | 0.81 ± 0.28 | 0.90 |
| K1 OD [D] | 45.18 ± 4.87 | 43.90 | 46.13 ± 6.31 | 44.0 |
| K2 OD [D] | 47.85 ± 5.99 | 46.10 | 49.07 ± 6.73 | 47.20 |
| Kmax OD [D] | 53.68 ± 9.32 | 51.30 | 55.02 ± 10.61 | 51.40 |
| anterior elevation OD [μm] | 23.14 ± 18.92 | 17.00 | 25.43 ± 16.27 | 24.00 |
| posterior elevation OD [μm] | 49.17 ± 36.42 | 39.00 | 58.05 ± 44.50 | 61.00 |
| TCT OD [µm] | 466.39 ± 54.28 | 473.50 | 460.62 ± 64.50 | 485.00 |
| thinnest epithelial thickness OD [μm] | 43.09 ± 5.45 | 44.00 | 43.50 ± 5.55 | 44.00 |
| K1 OS [D] | 45.54 ± 4.94 | 43.65 | 44.96 ± 3.43 | 44.40 |
| K2 OS [D] | 48.41 ± 5.91 | 46.45 | 47.84 ± 4.16 | 46.50 |
| Kmax OS [D] | 54.76 ± 9.49 | 52.15 | 53.51 ± 7.56 | 51.50 |
| anterior elevation OS [μm] | 26.44 ± 19.02 | 23.50 | 24.55 ± 15.42 | 20.50 |
| posterior elevation OS [μm] | 52.82 ± 33.64 | 50.00 | 52.25 ± 27.45 | 46.50 |
| TCT OS [µm] | 469.76 ± 54.66 | 473.50 | 474.80 ± 49.10 | 485.00 |
| thinnest epithelial thickness OS [μm] | 43.28 ± 5.44 | 44.00 | 44.57 ± 6.68 | 46.00 |
| AL OD [mm] | 24.03 ± 0.88 | 24.00 | 23.86 ± 0.91 | 23.60 |
| AL OS [mm] | 23.96 ± 0.85 | 23.90 | 23.76 ± 1.03 | 23.55 |
| IOP OD [mmHg] | 13.26 ± 3.16 | 13.00 | 12.72 ± 3.30 | 12.00 |
| IOP OS [mmHg] | 13.16 ± 3.30 | 13.00 | 12.94 ± 3.35 | 12.50 |

Abbreviations and symbols in the Table: x – average, SD – standard deviation, OD – oculus dexter, OS - oculus sinister, UDVA – uncorrected distance visual acuity, BDVA – best-corrected distance visual acuity, K1 – flat keratometric readings, K2 – steep keratometric readings, Kmax – maximum simulated keratometry, TCT – thinnest corneal thickness, AL - Axial length, IOP – intraocular pressure
