# Supplementary material for: Non-allergic eye rubbing is a major behavioral risk factor for keratoconus
Source: PLoS One. 2023 Apr 13;18(4):e0284454. doi: 10.1371/journal.pone.0284454 (PMC10101517; doi:10.1371/journal.pone.0284454)
Supplement: S2 Table — (DOCX) [file pone.0284454.s004.docx]

**S2 Table. Results of clinical comparison of adult and adolescent patients with KTCN**

|  | **Adults with KTCN (n=101)** | | **Adolescents with KTCN (n=17)** | |
| --- | --- | --- | --- | --- |
|  | **x** $\boldsymbol{\pm}$ **SD** | **Median** | **x**$\boldsymbol{\pm}$ **SD** | **Median** |
| UDVA OD | 0.47 ± 0.37 | 0.40 | 0.37 ± 0.48 | 0.40 |
| BDVA OD | 0.78 ± 0.35 | 0.90 | 0.88 ± 0.33 | 1.00 |
| UDVA OS | 0.46 ± 0.35 | 0.30 | 0.35 ± 0.32 | 0.20 |
| BDVA OS | 0.79 ± 0.37 | 0.90 | 0.73 ± 0.34 | 0.70 |
| K1 OD [D] | 45.42 ± 5.23 | 44.00 | 44.96 ± 4.76 | 43.60 |
| K2 OD [D] | 48.12 ± 6.04 | 46.60 | 47.77 ± 6.71 | 45.40 |
| Kmax OD [D] | 54.10 ± 9.40 | 51.90 | 55.93 ± 10.53 | 49.60 |
| anterior elevation OD [μm] | 23.89 ± 17.97 | 20.00 | 21.82 ± 21.39 | 15.00 |
| posterior elevation OD [μm] | 52.15 ± 37.63 | 46.00 | 42.77 ± 40.00 | 33.00 |
| TCT OD [µm] | 463.74 ± 56.64 | 470.50 | 474.59 ± 52.97 | 478.00 |
| thinnest epithelial thickness OD [μm] | 43.31 ± 5.72 | 44.00 | 42.25 ± 3.30 | 42.50 |
| K1 OS [D] | 45.40 ± 3.88 | 43.70 | 45.75 ± 3.64 | 44.30 |
| K2 OS [D] | 48.13 ± 5.76 | 46.40 | 49.40 ± 6.01 | 46.80 |
| Kmax OS [D] | 54.10 ± 9.17 | 52.10 | 57.11 ± 8.93 | 55.20 |
| anterior elevation OS [μm] | 25.98 ± 18.64 | 22.00 | 26.88 ± 17.45 | 24.00 |
| posterior elevation OS [μm] | 53.24 ± 33.18 | 50.00 | 49.71 ± 29.30 | 46.00 |
| TCT OS [µm] | 472.16 ± 53.30 | 478.00 | 462.00 ± 55.81 | 462.00 |
| thinnest epithelial thickness OS [μm] | 43.97 ± 5.61 | 45.00^†^ | 40.88 ± 5.48 | 42.00 |
| AL OD [mm] | 23.93 ± 0.83 | 23.90 | 24.34 ± 1.11 | 24.35 |
| AL OS [mm] | 23.87 ± 0.84 | 23.80 | 24.21 ± 1.04 | 24.15 |
| IOP OD [mmHg] | 13.00 ± 3.13 | 13.00 | 14.31 ± 3.35 | 13.00 |
| IOP OS [mmHg] | 13.12 ± 3.36 | 13.00 | 13.13 ± 3.02 | 13.00 |

Abbreviations and symbols in the Table: x – average, SD – standard deviation, OD – oculus dexter, OS - oculus sinister, UDVA – uncorrected distance visual acuity, BDVA – best-corrected distance visual acuity, K1 – flat keratometric readings, K2 – steep keratometric readings, Kmax – maximum simulated keratometry, TCT – thinnest corneal thickness, AL - Axial length, IOP – intraocular pressure

^†^ indicates statistically significant differences between KTCN patients subgroups with p-value ≤0.05

| **Variables** | | | **KTCN (n=118)** | **Control (n=73)** | **p-value** |  |
| --- | --- | --- | --- | --- | --- | --- |
| Age (years), mean±SD | | | 27.3 ± 8.7 | 31.5 ± 10.7 | 0.004 |  |
| Sex | | |  |  | < 0.001 |  |
|  | | Female | 21 (17.78%) | 42 (57.53%) |  |  |
|  | | Male | 97 (82.20%) | 31 (42.47%) |  |  |
| Level of education | | |  |  | 0.002 |  |
|  | | Primary | 14 (11.87%) | 2 (2.74%) |  |  |
|  | | Vocational education | 11 (9.32%) | 1 (1.37%) |  |  |
|  | | High school | 51 (43.22%) | 27 (36. 99%) |  |  |
|  | | University | 42 (35.59%) | 43 (58.90%) |  |  |
| Place of living up to the age of 15 | | |  |  | 0.004 |  |
|  | | Village | 43 (36.44%) | 32 (44.44%) |  |  |
|  | | City up to 20000 | 19 (16.10%) | 17 (23.61%) |  |  |
|  | | City from 20000 to 100000 | 7 (5.93%) | 11 (11.11%) |  |  |
|  | | City from 100000 to 500000 | 30 (25.43%) | 3 (4.17%) |  |  |
|  | | City with over 500000 | 19 (16.10%) | 12 (16.67%) |  |  |
| Allergy |  | |  |  | 0.128 |  |
|  | Yes | | 45 (38.14%) | 20 (27.40%) |  |  |
|  | No | | 73 (61.86%) | 53 (72.60%) |  |  |
| Food Allergy |  | |  |  | 0.223 |  |
|  | Yes | | 8 (6.78%) | 2 (2.74%) |  |  |
|  | No | | 110 (93.22%) | 71 (97.26%) |  |  |
| Pollen/grass/dust Allergy | | |  |  | 0.114 |  |
|  | Yes | | 42 (35.59%) | 18 (24.66%) |  |  |
|  | No | | 76 (64.41%) | 55 (75.34%) |  |  |
| Professional occupation | |  |  |  | 0.128 |  |
|  | | Student | 27 (23.68%) | 9 (12.50%) |  |  |
|  | | Non-office worker | 55 (48.25%) | 36 (50.00%) |  |  |
|  | | Office worker | 32 (28.07%) | 27 (37.50%) |  |  |
| Dust in the working environment | | |  |  | 0.016 |  |
|  | | Yes | 34 (28.81%) | 10 (13.70%) |  |  |
|  | | No | 84 (71.19%) | 63 (86.30%) |  |  |
| Using a computer at work (hours per day) | | | 5.7±2.8 | 6.1±2.3 | 0.617 |  |
| Using a computer after work (hours per day) | | | 2.4±1.2 | 1.6±0.9 | < 0.001 |  |
| Eye rubbing | | |  |  |  | < 0.001 |
|  | | | Yes | 92.373% (109) | 50 (68.493%) |  |
|  | | | No | 7.627% (9) | 23 (31.507%) |  |
| Frequent eye rubbing | | |  |  |  | 0.011 |
|  | | | Yes | 10 (8.48%) | 0 (0.00%) |  |
|  | | | No | 108 (91.52%) | 73 (100.00%) |  |
| Dominant hand | | |  |  | 0.006 |  |
|  | | | Right | 100 (84.75%) | 71 (97.26%) |  |
|  | | | Left | 18 (15.25%) | 2 (2.74%) |  |
|  | | |  |  |  |  |

|  |  | |  |  |  |
| --- | --- | --- | --- | --- | --- |
|  |  | |  |  |  |
|  |  | |  |  |  |
|  |  | |  |  |  |
|  |  | |  |  |  |
|  |  | |  |  |  |
|  | | |  |  |  |
|  |  | |  |  |  |
|  |  | |  |  |  |
| More frequently rubbed eye | | |  |  | 0.020 |
|  | Both | | 79 (72.48%) | 44 (1.67%) |  |
|  | Right | | 14 (12.84%) | 3 (6.25%) |  |
|  | Left | | 16 (14.68%) | 1 (2.08%) |  |
| Part of the hand used for rubbing | | |  |  | 0.052 |
|  | | Fingertips | 37 (41.11%) | 23 (48.94%) |  |
|  | | Base of hand | 2 (2.22%) | 3 (6.38%) |  |
|  | | Knuckles | 29 (32.22%) | 18 (38.30%) |  |
|  | | Fists | 22 (24.45%) | 3 (6.38%) |  |
| Eye rubbing with a fist | |  |  |  | 0.009 |
|  | | Yes | 22 (24.44%) | 3 (6.38%) |  |
|  | | No | 68 (75.56%) | 44 (93.62%) |  |
| The upper eyelid as the most frequently rubbed part | | |  |  | 0.033 |
|  | | Yes | 45 (38.14%) | 17 (23.29%) |  |
|  | | No | 73 (61.86%) | 56 (76.71%) |  |
| The lower eyelid as the most frequently rubbed part | | |  |  | 0.004 |
|  | | Yes | 40 (33.90%) | 11 (15.07%) |  |
|  | | No | 78 (66.10%) | 62 (84.93%) |  |
| Type of eye rubbing indicated in response to presented photographs | | |  |  | 0.075 |
|  | | Photography no. 1 | 11 (11.96%) | 14 (28.00%) |  |
|  | | Photography no. 2 | 14 (15.22%) | 11 (22.00%) |  |
|  | | Photography no. 3 | 9 (9.78%) | 4 (8.00%) |  |
|  | | Photography no. 4 | 7 (7.61%) | 7 (14.00%) |  |
|  | | Photography no. 5 | 10 (10.87%) | 4 (8.00%) |  |
|  | | Photography no. 6 | 9 (9.78%) | 2 (4.00%) |  |
|  | | Photography no. 7 | 6 (6.52%) | 2 (4.00%) |  |
|  | | Photography no. 8 | 26 (28.26%) | 6 (12.00%) |  |
| Photographs no. 1-4 or 5-8 | | |  |  | 0.002 |
|  | | Photography 1 or 2 or 3 or 4 | 41 (44.56%) | 36 (72.00%) |  |
|  | | Photography 5 or 6 or 7 or 8 | 51 (55.44%) | 14 (28.00%) |  |
| Rubbing the eyes immediately after waking up | | |  |  | 0.191 |
|  | | Yes | 50 (42.37%) | 24 (32.88%) |  |
|  | | No | 68 (57.63%) | 49 (67.12%) |  |

Note: the respondents did not always answer all the questions, therefore the summation of the answers in individual questions in the table may be incomplete.
