# Supplementary material for: Non-allergic eye rubbing is a major behavioral risk factor for keratoconus
Source: PLoS One. 2023 Apr 13;18(4):e0284454. doi: 10.1371/journal.pone.0284454 (PMC10101517; doi:10.1371/journal.pone.0284454)
Supplement: S3 Table — (DOCX) [file pone.0284454.s005.docx]

**S3 Table**. **Complete information on the qualitative and quantitative data/results of analyzes of behavioral, environmental, and socioeconomic aspects**

| **Variables** | | **KTCN (n=118)** | **Control (n=73)** | **p-value** |
| --- | --- | --- | --- | --- |
| Age (years), mean±SD | | 27.3 ± 8.7 | 31.5 ± 10.7 | 0.004 |
| Sex | |  |  | < 0.001 |
|  | Female | 21 (17.78%) | 42 (57.53%) |  |
|  | Male | 97 (82.20%) | 31 (42.47%) |  |
| Level of education | |  |  | 0.002 |
|  | Primary | 14 (11.87%) | 2 (2.74%) |  |
|  | Vocational education | 11 (9.32%) | 1 (1.37%) |  |
|  | High school | 51 (43.22%) | 27 (36. 99%) |  |
|  | University | 42 (35.59%) | 43 (58.90%) |  |
| Place of living up to the age of 15 | |  |  | 0.004 |
|  | Village | 43 (36.44%) | 32 (44.44%) |  |
|  | City up to 20000 residents | 19 (16.10%) | 17 (23.61%) |  |
|  | City from 20000 to 100000 residents | 7 (5.93%) | 11 (11.11%) |  |
|  | City from 100000 to 500000 residents | 30 (25.43%) | 3 (4.17%) |  |
|  | City with over 500000 residents | 19 (16.10%) | 12 (16.67%) |  |
| Time of outdoor physical activity up to the age of 15 (hours per day) | | 2.5 ± 1.2 | 2.5 ± 1.1 | 0.421 |
| Vision correction |  |  |  | 0.077 |
|  | glasses | 59 (50.00%) | 23 (31.51%) |  |
|  | soft lenses | 2 (1.70%) | 3 (4.11%) |  |
|  | hard lenses | 1 (0.85%) | 0 (0%) |  |
|  | lenses and glasses | 3 (2.55%) | 2 (2.74%) |  |
|  | No correction | 53 (44.92%) | 45 (61.64%) |  |
| Conjunctivitis |  |  |  | 0.087 |
|  | Yes | 20 (16.95%) | 6 (8.22%) |  |
|  | No | 98 (83.05%) | 67 (91.78%) |  |
| Dry eye syndrome |  |  |  | 0.432 |
|  | Yes | 6 (5.09%) | 2 (2.74%) |  |
|  | No | 112 (94.91%) | 71 (97.26%) |  |
| Eye trauma |  |  |  | 0.592 |
|  | Yes | 5 (4.24%) | 2 (2.74%) |  |
|  | No | 113 (95.76%) | 71 (97.26%) |  |
| Allergy |  |  |  | 0.128 |
|  | Yes | 45 (38.14%) | 20 (27.40%) |  |
|  | No | 73 (61.86%) | 53 (72.60%) |  |
| Food allergy |  |  |  | 0.223 |
|  | Yes | 8 (6.78%) | 2 (2.74%) |  |
|  | No | 110 (93.22%) | 71 (97.26%) |  |
| Pollen/grass/dust allergy | |  |  | 0.114 |
|  | Yes | 42 (35.59%) | 18 (24.66%) |  |
|  | No | 76 (64.41%) | 55 (75.34%) |  |
| Asthma |  |  |  | 0.44 |
|  | Yes | 10 (8.47%) | 6 (5.48%) |  |
|  | No | 108 (91.53%) | 69 (94.52%) |  |
| Atopic dermatitis |  |  |  | 0.396 |
|  | Yes | 4 (3.39%) | 1 (1.37%) |  |
|  | No | 114 (96.61%) | 72 (98.63%) |  |
| Thyroid gland diseases |  |  |  | 0.386 |
|  | Yes | 6 (5.09%) | 6 (8.22%) |  |
|  | No | 112 (94.91%) | 67 (91.78%) |  |
| Smoking |  |  |  | 0.686 |
|  | Yes | 23 (19.49%) | 16 (21.92%) |  |
|  | No | 95 (80.51%) | 57 (78.08%) |  |
| Eye rubbing |  |  |  | < 0.001 |
|  | Yes | 92.373% (109) | 50 (68.493%) |  |
|  | No | 7.627% (9) | 23 (31.507%) |  |
| Frequent eye rubbing |  |  |  | 0.011 |
|  | Yes | 10 (8.48%) | 0 (0.00%) |  |
|  | No | 108 (91.52%) | 73 (100.00%) |  |
| Dominant hand | |  |  | 0.006 |
|  | Right | 100 (84.75%) | 71 (97.26%) |  |
|  | Left | 18 (15.25%) | 2 (2.74%) |  |
| More frequently rubbed eye | |  |  | 0.020 |
|  | Both | 79 (72.48%) | 44 (1.67%) |  |
|  | Right | 14 (12.84%) | 3 (6.25%) |  |
|  | Left | 16 (14.68%) | 1 (2.08%) |  |
| Part of the hand used for rubbing | |  |  | 0.052 |
|  | Fingertips | 37 (41.11%) | 23 (48.94%) |  |
|  | Base of hand | 2 (2.22%) | 3 (6.38%) |  |
|  | Knuckles | 29 (32.22%) | 18 (38.30%) |  |
|  | Fists | 22 (24.45%) | 3 (6.38%) |  |
| Eye rubbing with a fist |  |  |  | 0.009 |
|  | Yes | 22 (24.44%) | 3 (6.38%) |  |
|  | No | 68 (75.56%) | 44 (93.62%) |  |
| The inner corner of the eye as the most frequently rubbed part | |  |  | 0.204 |
|  | Yes | 58 (49.15%) | 29 (39.73%) |  |
|  | No | 60 (50.85%) | 44 (60.27%) |  |
| The outer corner of the eye as the most frequently rubbed part | |  |  | 0.141 |
|  | Yes | 32 (27.12%) | 13 (17.81%) |  |
|  | No | 86 (72.88%) | 60 (82.19%) |  |
| The upper eyelid as the most frequently rubbed part | |  |  | 0.033 |
|  | Yes | 45 (38.14%) | 17 (23.29%) |  |
|  | No | 73 (61.86%) | 56 (76.71%) |  |
| The lower eyelid as the most frequently rubbed part | |  |  | 0.004 |
|  | Yes | 40 (33.90%) | 11 (15.07%) |  |
|  | No | 78 (66.10%) | 62 (84.93%) |  |
| Type of eye rubbing indicated in response to presented photographs | |  |  | 0.075 |
|  | Photography no. 1 | 11 (11.96%) | 14 (28.00%) |  |
|  | Photography no. 2 | 14 (15.22%) | 11 (22.00%) |  |
|  | Photography no. 3 | 9 (9.78%) | 4 (8.00%) |  |
|  | Photography no. 4 | 7 (7.61%) | 7 (14.00%) |  |
|  | Photography no. 5 | 10 (10.87%) | 4 (8.00%) |  |
|  | Photography no. 6 | 9 (9.78%) | 2 (4.00%) |  |
|  | Photography no. 7 | 6 (6.52%) | 2 (4.00%) |  |
|  | Photography no. 8 | 26 (28.26%) | 6 (12.00%) |  |
| Photographs no. 1-4 or 5-8 | |  |  | 0.002 |
|  | Photography 1 or 2 or 3 or 4 | 41 (44.56%) | 36 (72.00%) |  |
|  | Photography 5 or 6 or 7 or 8 | 51 (55.44%) | 14 (28.00%) |  |
| Sleeping Position |  |  |  | 0.990 |
|  | Back | 6 (6.32%) | 4 (5.88%) |  |
|  | Belly | 20 (21.05%) | 14 (20.59%) |  |
|  | Side | 69 (72.63%) | 50 (73.53%) |  |
| Head orientation while sleeping | |  |  | 0.978 |
|  | Right | 34 (61.82%) | 24 (61.54%) |  |
|  | Left | 21 (38.18%) | 15 (38.46%) |  |
| Hand/arm position while sleeping | |  |  | 0.864 |
|  | Under head | 10 (35.71%) | 8 (38.09%) |  |
|  | Under pillow | 18 (64.29%) | 13 (61.91%) |  |
| Rubbing the eyes immediately after waking up | |  |  | 0.191 |
|  | Yes | 50 (42.37%) | 24 (32.88%) |  |
|  | No | 68 (57.63%) | 49 (67.12%) |  |
| Professional occupation |  |  |  | 0.128 |
|  | Student | 27 (23.68%) | 9 (12.50%) |  |
|  | Non-office worker | 55 (48.25%) | 36 (50.00%) |  |
|  | Office worker | 32 (28.07%) | 27 (37.50%) |  |
| Excessive UVA exposure | |  |  | 0.431 |
|  | Yes | 5 (4.24%) | 5 (6.85%) |  |
|  | No | 113 (95.76%) | 68 (93.15%) |  |
| Dust in the working environment | |  |  | 0.016 |
|  | Yes | 34 (28.81%) | 10 (13.70%) |  |
|  | No | 84 (71.19%) | 63 (86.30%) |  |
| Sports activities as a hobby, up to the age of 18 | |  |  | 0.179 |
|  | Yes | 49 (53.85%) | 42 (64.61%) |  |
|  | No | 42 (46.15%) | 23 (35.39%) |  |
| Usage of electronics as a hobby, up to the age of 18 | |  |  | 0.101 |
|  | Yes | 17 (18.68%) | 6 (9.23%) |  |
|  | No | 74 (81.32%) | 59 (90.77%) |  |
| Book reading as a hobby, up to the age of 18 | |  |  | 0.611 |
|  | Yes | 14 (15.38%) | 12 (18.46%) |  |
|  | No | 77 (84.62%) | 53 (81.54%) |  |
| Sports activities as a hobby, over the age of 18 | |  |  | 0.281 |
|  | Yes | 39 (42.86%) | 35 (51.47%) |  |
|  | No | 52 (57.14%) | 33 (48.53%) |  |
| Usage of electronics as a hobby, over the age of 18 | |  |  | 0.797 |
|  | Yes | 7 (7.69%) | 6 (8.82%) |  |
|  | No | 84 (92.31%) | 62 (91.18%) |  |
| Book reading as a hobby, over the age of 18 | |  |  | 0.829 |
|  | Yes | 11 (12.09%) | 9 (13.24%) |  |
|  | No | 80 (87.91%) | 59 (86.77%) |  |
| Professional work on the computer | |  |  | 0.021 |
|  | Yes | 69 (58.97%) | 55 (75.34%) |  |
|  | No | 48 (41.03%) | 18 (24.66%) |  |
| Using a computer at work (hours per day) | | 5.7±2.8 | 6.1±2.3 | 0.617 |
| Using a computer after work (hours per day) | | 2.4±1.2 | 1.6±0.9 | < 0.001 |
| Work or study at night (hours per day) | | 1.2±0.7 | 1.1±0.5 | 0.279 |
| Work or study at night on the computer (hours per day) | | 0.9±0.6 | 0.7±0.5 | 0.204 |
| Reading paper books or newspapers | |  |  | < 0.001 |
|  | Yes | 70 (59.83%) | 61 (83.56%) |  |
|  | No | 47 (40.17%) | 12 (16.44%) |  |
| Reading from electronic readers | |  |  | 0.045 |
|  | Yes | 39 (33.33%) | 35 (47.94%) |  |
|  | No | 78 (66.67%) | 38 (52.06%) |  |
| Reading in artificial light | |  |  | < 0.001 |
|  | Yes | 79 (67.52%) | 65 (89.04%) |  |
|  | No | 38 (32.48%) | 8 (10.96%) |  |
| Using a smartphone |  |  |  | 0.125 |
|  | Yes | 110 (94.02%) | 64 (87.67%) |  |
|  | No | 7 (5.98%) | 9 (12.33%) |  |
| Using a smartphone (hours per day) | | 1.2±0.9 | 1.4±0.9 | 0.071 |
| Using a smartphone at dark | |  |  | 0.671 |
|  | Yes | 75 (64.103%) | 49 (67.123%) |  |
|  | No | 42 (35.897%) | 24 (32.877%) |  |
| Using a smartphone at dark (hours per day) | | 0.7±0.8 | 0.7±0.7 | 0.041 |

Note: the respondents did not always answer all the questions, therefore the summation of the answers in individual questions in the table may be incomplete.
