# Supplementary material for: Non-allergic eye rubbing is a major behavioral risk factor for keratoconus
Source: PLoS One. 2023 Apr 13;18(4):e0284454. doi: 10.1371/journal.pone.0284454 (PMC10101517; doi:10.1371/journal.pone.0284454)
Supplement: S4 Table — (DOCX) [file pone.0284454.s006.docx]

**S4 Table. Results of comparison of male and female patients with KTCN in aspect of selected behavioral, environmental, and socioeconomic factors.**

| **Variables** |  | **Males with KTCN (n=97)** | **Females with KTCN (n=21)** | **p-value** |
| --- | --- | --- | --- | --- |
| Age (years), mean±SD |  | 27.70 ± 8.79 | 25.71 ± 8.20 | 0.347 |
| Subgroup |  |  |  | 0.504 |
|  | Adults with KTCN | 84 (86.59%) | 17 (80.95%) |  |
|  | Adolescents with KTCN | 13 (13.40%) | 4 (19.05%) |  |
| Level of education |  |  |  | 0.263 |
|  | Primary | 11 (11.34%) | 3 (14.29%) |  |
|  | Vocational education | 10 (10.31%) | 1 (4.76%) |  |
|  | High school | 45 (46.39%) | 6 (28.57%) |  |
|  | University | 31 (31.96%) | 11 (52.38%) |  |
| Place of living up to the age of 15 | |  |  | 0.072 |
|  | Village | 31 (31.96%) | 12 (57.14%) |  |
|  | City up to 20000 residents | 14 (14.43%) | 5 (23.81%) |  |
|  | City from 20000 to 100000 residents | 6 (6.19%) | 1 (4.76%) |  |
|  | City from 100000 to 500000 residents | 28 (28.87%) | 2 (9.52%) |  |
|  | City with over 500000 residents | 18 (18.56%) | 1 (4.76%) |  |
| Allergy |  |  |  | 0.617 |
|  | Yes | 38 (39.18%) | 7 (33.33%) |  |
|  | No | 59 (60. 82%) | 14 (66.67%) |  |
| Food Allergy |  |  |  | 0.685 |
|  | Yes | 7 (7.22%) | 1 (4.76%) |  |
|  | No | 90 (92.78%) | 20 (95.24%) |  |
| Pollen/grass/dust Allergy |  |  |  | 0.459 |
|  | Yes | 36 (37.11%) | 6 (28.57%) |  |
|  | No | 61 (62.89%) | 15 (71.43%) |  |
| Professional occupation |  |  |  | 0.426 |
|  | Student | 21 (22.34%) | 6 (30.00%) |  |
|  | Non-office worker | 48 (51.06%) | 7 (35.00%) |  |
|  | Office worker | 25 (26.60%) | 7 (35.00%) |  |
| Dust in the working environment | |  |  | 0.031 |
|  | Yes | 32 (32.99%) | 2 (9.52%) |  |
|  | No | 65 (67.01%) | 19 (90.48%) |  |
| Using a computer at work/study (hours per day) | | 5.6±3.0 | 6.4±2.1 | 0.330 |
| Using a computer after work/study (hours per day) | | 2.4±1.2 | 2.4±1.2 | 0.897 |
| Eye rubbing |  |  |  | 0.718 |
|  | Yes | 90 (92.78%) | 19 (90.48%) |  |
|  | No | 7 (7.22%) | 2 (9.52%) |  |
| Frequent eye rubbing |  |  |  | 0.849 |
|  | Yes | 8 (8.25%) | 2 (9.52%) |  |
|  | No | 89 (91.75%) | 19 (90.48%) |  |
| Dominant hand |  |  |  | 0.140 |
|  | Right | 80 (82.47%) | 20 (95.24%) |  |
|  | Left | 17 (17.53%) | 1 (4.76%) |  |
|  |  |  |  |  |
| More frequently rubbed eye |  |  |  | 0.150 |
|  | Both | 67 (74.44%) | 12 (63.16%) |  |
|  | Right | 9 (10.00%) | 5 (26.32%) |  |
|  | Left | 14 (15.56%) | 2 (10.53%) |  |
| Part of the hand used for rubbing | |  |  | 0.699 |
|  | Fingertips | 32 (43.24%) | 5 (31.25%) |  |
|  | Base of hand | 2 (2.70%) | 0 (0.00%) |  |
|  | Knuckles | 23 (31.08%) | 6 (37.50%) |  |
|  | Fists | 17 (22.97%) | 5 (31.25%) |  |
| Eye rubbing with a fist |  |  |  | 0.485 |
|  | Yes | 17 (22.97%) | 5 (31.25%) |  |
|  | No | 57 (77.03%) | 11 (68.75%) |  |
| The upper eyelid as the most frequently rubbed part | |  |  | 0.865 |
|  | Yes | 36 (40.00%) | 8 (42.11%) |  |
|  | No | 54 (60.00%) | 11 (57.89%) |  |
| The lower eyelid as the most frequently rubbed part | |  |  | 0.344 |
|  | Yes | 34 (37.78%) | 5 (26.32%) |  |
|  | No | 56 (62.22%) | 14 (73.68%) |  |
| Type of eye rubbing indicated in response to presented photographs | | |  | 0.051 |
|  | Photography no. 1 | 8 (10.53%) | 3 (18.75%) |  |
|  | Photography no. 2 | 8 (10.53%) | 6 (37.50%) |  |
|  | Photography no. 3 | 7 (9.21%) | 2 (12.50%) |  |
|  | Photography no. 4 | 7 (9.21%) | 0 (0.00%) |  |
|  | Photography no. 5 | 10 (13.16%) | 0 (0.00%) |  |
|  | Photography no. 6 | 9 (11.84%) | 0 (0.00%) |  |
|  | Photography no. 7 | 6 (7.90%) | 0 (0.00%) |  |
|  | Photography no. 8 | 21 (27.63%) | 5 (31.25%) |  |
| Photographs no. 1-4 or 5-8 |  |  |  | 0.032 |
|  | Photography 1 or 2 or 3 or 4 | 30 (39.47%) | 11 (68.75%) |  |
|  | Photography 5 or 6 or 7 or 8 | 46 (60.53%) | 5 (31.25%) |  |
| Rubbing the eyes immediately after waking up | |  |  | 0.306 |
|  | Yes | 11 (52.38%) | 39 (40.21%) |  |
|  | No | 10 (47.62%) | 58 (59.79%) |  |
