# Supplementary material for: Non-allergic eye rubbing is a major behavioral risk factor for keratoconus
Source: PLoS One. 2023 Apr 13;18(4):e0284454. doi: 10.1371/journal.pone.0284454 (PMC10101517; doi:10.1371/journal.pone.0284454)
Supplement: S5 Table — (DOCX) [file pone.0284454.s007.docx]

**S5 Table. Results of comparison of adult and adolescent patients with KTCN in aspect of selected behavioral, environmental, and socioeconomic factors.**

| **Variables** |  | **Adults with KTCN (n=101)** | **Adolescents with KTCN (n=17)** | **p-value** |
| --- | --- | --- | --- | --- |
| Age (years), mean±SD |  | 29.19 ± 7.99 | 16.35 ± 1.94 | < 0.001 |
| Sex |  |  |  | 0.504 |
|  | Female | 17 (16.83%) | 4 (23.53%) |  |
|  | Male | 84 (83.17%) | 13 (76.47%) |  |
| Level of education |  |  |  | < 0.001 |
|  | Primary | 2 (1.98%) | 12 (70.59%) |  |
|  | Vocational education | 10 (9.9%) | 1 (1.37%) |  |
|  | High school | 47 (46.54%) | 4 (23.53%) |  |
|  | University | 42 (41.58%) | 0 (0.00%) |  |
| Place of living up to the age of 15 | |  |  | 0.435 |
|  | Village | 35 (34.65%) | 8 (47.06%) |  |
|  | City up to 20000 residents | 18 (17.82%) | 1 (5.88%) |  |
|  | City from 20000 to 100000 residents | 7 (6.93%) | 0 (0.00%) |  |
|  | City from 100000 to 500000 residents | 26 (25.74%) | 4 (23.53%) |  |
|  | City with over 500000 residents | 15 (14.85%) | 4 (23.53%) |  |
| Allergy |  |  |  | 0.423 |
|  | Yes | 40 (39.60%) | 5 (29.41%) |  |
|  | No | 61 (60.40%) | 12 (70.59%) |  |
| Food Allergy |  |  |  | 0.874 |
|  | Yes | 7 (6.93%) | 1 (5.88%) |  |
|  | No | 94 (93.07%) | 16 (94.12%) |  |
| Pollen/grass/dust Allergy |  |  |  | 0.565 |
|  | Yes | 37 (36.63%) | 5 (29.41%) |  |
|  | No | 64 (63.37%) | 12 (70.59%) |  |
| Professional occupation |  |  |  | < 0.001 |
|  | Student | 10 (10.31%) | 17 (100.00%) |  |
|  | Non-office worker | 55 (56.70%) | 0 (0.00%) |  |
|  | Office worker | 32 (32.99%) | 0 (0.00%) |  |
| Dust in the working environment | |  |  | 0.024 |
|  | Yes | 33 (32.67%) | 1 (5.88%) |  |
|  | No | 68 (67.33%) | 16 (94.12%) |  |
| Using a computer at work/study (hours per day) | | 6.1±2.8 | 3.3±1.6 | 0.008 |
| Using a computer after work/study (hours per day) | | 2.3±1.2 | 2.4±1.2 | 0.871 |
| Eye rubbing |  |  |  | 0.770 |
|  | Yes | 93 (92.08%) | 16 (94.12%) |  |
|  | No | 8 (7.92%) | 1 (5.88%) |  |
| Frequent eye rubbing |  |  |  | 0.678 |
|  | Yes | 9 (8.91%) | 1 (5.88%) |  |
|  | No | 92 (91.09%) | 16 (94.12%) |  |
| Dominant hand |  |  |  | 0.665 |
|  | Right | 85 (84.16%) | 15 (88.24%) |  |
|  | Left | 16 (15.84%) | 2 (11.76%) |  |
|  |  |  |  |  |
| More frequently rubbed eye |  |  |  | 0.106 |
|  | Both | 64 (68.82%) | 15 (93.75%) |  |
|  | Right | 14 (15.05%) | 1 (6.25%) |  |
|  | Left | 15 (16.13%) | 0 (0.00%) |  |
| Part of the hand used for rubbing | |  |  | 0.413 |
|  | Fingertips | 33 (41.77%) | 4 (36.36%) |  |
|  | Base of hand | 1 (1.27%) | 1 (9.09%) |  |
|  | Knuckles | 26 (32.91%) | 3 (27.27%) |  |
|  | Fists | 19 (24.05%) | 3 (27.27%) |  |
| Eye rubbing with a fist |  |  |  | 0.816 |
|  | Yes | 19 (24.05%) | 3 (27.27%) |  |
|  | No | 60 (75.95%) | 8 (72.73%) |  |
| The upper eyelid as the most frequently rubbed part | |  |  | 0.421 |
|  | Yes | 39 (41.93%) | 5 (31.25%) |  |
|  | No | 54 (58.07%) | 11 (68.75%) |  |
| The lower eyelid as the most frequently rubbed part | |  |  | 0.330 |
|  | Yes | 35 (37.63%) | 4 (25.00%) |  |
|  | No | 58 (62.37%) | 12 (75.00%) |  |
| Type of eye rubbing indicated in response to presented photographs | | |  | 0.664 |
|  | Photography no. 1 | 10 (12.35%) | 1 (9.09%) |  |
|  | Photography no. 2 | 14 (17.28%) | 0 (0.00%) |  |
|  | Photography no. 3 | 8 (9.88%) | 1 (9.09%) |  |
|  | Photography no. 4 | 6 (7.41%) | 1 (9.09%) |  |
|  | Photography no. 5 | 9 (11.11%) | 1 (9.09%) |  |
|  | Photography no. 6 | 8 (9.88%) | 1 (9.09%) |  |
|  | Photography no. 7 | 4 (4.94%) | 2 (18.18%) |  |
|  | Photography no. 8 | 22 (27.16%) | 4 (36.36%) |  |
| Photographs no. 1-4 or 5-8 |  |  |  | 0.219 |
|  | Photography 1 or 2 or 3 or 4 | 38 (46.91%) | 3 (27.27%) |  |
|  | Photography 5 or 6 or 7 or 8 | 43 (53.07%) | 8 (72.73%) |  |
| Rubbing the eyes immediately after waking up | |  |  | 0.673 |
|  | Yes | 42 (41.58%) | 8 (47.06%) |  |
|  | No | 59 (58.42%) | 9 (52.94%) |  |
