# Supplementary material for: Non-allergic eye rubbing is a major behavioral risk factor for keratoconus
Source: PLoS One. 2023 Apr 13;18(4):e0284454. doi: 10.1371/journal.pone.0284454 (PMC10101517; doi:10.1371/journal.pone.0284454)
Supplement: S6 Table — (DOCX) [file pone.0284454.s008.docx]

**S6 Table. Additional statistical analyses**

1. Results of the relation between the presence of the dust in the working environment and eye rubbing (p-value=0.044)

|  | Eye rubbing | |
| --- | --- | --- |
|  | Yes | No |
| Dust in working environment |  |  |
| Yes | 41 (93.18%) | 3 (6.82%) |
| No | 118 (80.27%) | 29 (19.73%) |

|  | | | | | | Dominant hand | | | |  | |
| --- | --- | --- | --- | --- | --- | --- | --- | --- | --- | --- | --- |
| Group | | More frequently rubbed eye | |  | | Left | | Right | | Total | |
| Control |  | both |  | Count |  | 2.000 |  | 42.000 |  | 44.000 |  |
|  |  |  |  | % within column |  | 100.000 % |  | 91.304 % |  | 91.667 % |  |
|  |  | left |  | Count |  | 0.000 |  | 1.000 |  | 1.000 |  |
|  |  |  |  | % within column |  | 0.000 % |  | 2.174 % |  | 2.083 % |  |
|  |  | right |  | Count |  | 0.000 |  | 3.000 |  | 3.000 |  |
|  |  |  |  | % within column |  | 0.000 % |  | 6.522 % |  | 6.250 % |  |
|  |  | total |  | Count |  | 2.000 |  | 46.000 |  | 48.000 |  |
|  |  |  |  | % within column |  | 100.000 % |  | 100.000 % |  | 100.000 % |  |
| Keratoconus |  | both |  | Count |  | 13.000 |  | 66.000 |  | 79.000 |  |
|  |  |  |  | % within column |  | 76.471 % |  | 71.739 % |  | 72.477 % |  |
|  |  | left |  | Count |  | 3.000 |  | 13.000 |  | 16.000 |  |
|  |  |  |  | % within column |  | 17.647 % |  | 14.130 % |  | 14.679 % |  |
|  |  | right |  | Count |  | 1.000 |  | 13.000 |  | 14.000 |  |
|  |  |  |  | % within column |  | 5.882 % |  | 14.130 % |  | 12.844 % |  |
|  |  | total |  | Count |  | 17.000 |  | 92.000 |  | 109.000 |  |
|  |  |  |  | % within column |  | 100.000 % |  | 100.000 % |  | 100.000 % |  |
| Control&Keratoconus |  | both |  | Count |  | 15.000 |  | 108.000 |  | 123.000 |  |
|  |  |  |  | % within column |  | 78.947 % |  | 78.261 % |  | 78.344 % |  |
|  |  | left |  | Count |  | 3.000 |  | 14.000 |  | 17.000 |  |
|  |  |  |  | % within column |  | 15.789 % |  | 10.145 % |  | 10.828 % |  |
|  |  | right |  | Count |  | 1.000 |  | 16.000 |  | 17.000 |  |
|  |  |  |  | % within column |  | 5.263 % |  | 11.594 % |  | 10.828 % |  |
|  |  | total |  | Count |  | 19.000 |  | 138.000 |  | 157.000 |  |
|  |  |  |  | % within column |  | 100.000 % |  | 100.000 % |  | 100.000 % |  |
|  | | | | | | | | | | | |

1. Results of the relation between the most frequently rubbed eye and right / left-handedness

| Chi-Squared Tests | | | | | | | | | |  |
| --- | --- | --- | --- | --- | --- | --- | --- | --- | --- | --- |
| Group | |  | Value | | df | | | p | |  |
| Control |  | Χ² |  | 0.190 | |  | 2 |  | 0.909 |  |
|  |  | N |  | 48 | |  |  |  |  |  |
| Keratoconus |  | Χ² |  | 0.925 | |  | 2 |  | 0.630 |  |
|  |  | N |  | 109 | |  |  |  |  |  |
| Control&Keratoconus |  | Χ² |  | 1.111 | |  | 2 |  | 0.574 |  |
|  |  | N |  | 157 | |  |  |  |  |  |
|  | | | | | | | | | |  |

1. Results of the relation between the most frequently rubbed eye and the more affected eye

| Contingency Tables | | | | | | | | | |
| --- | --- | --- | --- | --- | --- | --- | --- | --- | --- |
|  | | | | More affected_eye | | | |  | |
| More frequently rubbed eye | |  | | OD | | OS | | Total | |
| Both |  | Count |  | 24.000 |  | 53.000 |  | 77.000 |  |
|  |  | % within column |  | 63.158 % |  | 76.812 % |  | 71.963 % |  |
| Left |  | Count |  | 3.000 |  | 13.000 |  | 16.000 |  |
|  |  | % within column |  | 7.895 % |  | **18.841 %** |  | 14.953 % |  |
| Right |  | Count |  | 11.000 |  | 3.000 |  | 14.000 |  |
|  |  | % within column |  | 28.947 % |  | 4.348 % |  | 13.084 % |  |
| Total |  | Count |  | 38.000 |  | 69.000 |  | 107.000 |  |
|  |  | % within column |  | 100.000 % |  | 100.000 % |  | 100.000 % |  |
|  | | | | | | | | | |

| Chi-Squared Tests | | | | | | |
| --- | --- | --- | --- | --- | --- | --- |
|  | | Value | | df | | p |
| Χ² |  | 13.932 |  | 2 |  | **< 0.001** |
| N |  | 107 |  |  |  |  |

1. Results of the relation between the eye rubbing and absolute differences in TCT (TCT diff) between right and left eye

| TCT diff | | | | | | | | | |
| --- | --- | --- | --- | --- | --- | --- | --- | --- | --- |
|  | | Test | | Statistic | | df | | p | |
|  |  | Mann-Whitney |  | 1714.000 |  |  |  | 0.004 |  |
|  | | | | | | | | | |

| TCT diff – a descriptive statistic | | | | | |
| --- | --- | --- | --- | --- | --- |
|  | | Eye rubbing | | | |
|  | | No | | Yes | |
| Valid |  | 32 |  | 159 |  |
| Missing |  | 0 |  | 0 |  |
| Mean |  | 12.563 |  | 37.642 |  |
| Std. Deviation |  | 18.746 |  | 67.323 |  |
| Minimum |  | 0.000 |  | 0.000 |  |
| Maximum |  | 76.000 |  | 526.000 |  |
|  | | | | | |

| TCT diff – logistic regression | | | | | | | | | | | | | | | |
| --- | --- | --- | --- | --- | --- | --- | --- | --- | --- | --- | --- | --- | --- | --- | --- |
|  | | | | | | | | | | Wald Test | | | | | |
|  | |  | |  | | Odds Ratio | | z | | Wald Statistic | | df | | p | |
|  |  |  |  |  |  |  |  |  |  |  |  |  |  |  |  |
| TCT_diff |  | 0.028 |  | 0.011 |  | 1.028 |  | 2.503 |  | 6.263 |  | 1 |  | 0.012 |  |
|  | | | | | | | | | | | | | | | |
| *Note.*  eye_rubbing level 'yes' coded as class 1. | | | | | | | | | | | | | | | |

1. Results of the relation between eye rubbing and allergy status

| Contingency Tables | | | | | | | |
| --- | --- | --- | --- | --- | --- | --- | --- |
|  | | Allergy | | | |  | |
| Eye_rubbing | | No | | Yes | | Total | |
| no |  | 6 |  | 3 |  | 9 |  |
| yes |  | 67 |  | 42 |  | 109 |  |
| Total |  | 73 |  | 45 |  | 118 |  |
|  | | | | | | | |

| Chi-Squared Tests | | | | | | | |
| --- | --- | --- | --- | --- | --- | --- | --- |
|  | | Value | | df | | p | |
| Χ² |  | 0.095 |  | 1 |  | 0.758 |  |
| N |  | 118 |  |  |  |  |  |
|  | | | | | | | |

| Contingency Tables | | | | | | | |
| --- | --- | --- | --- | --- | --- | --- | --- |
|  | | Pollen/grass/dust allergy | | | |  | |
| Part of hand used for rubbing | | No | | Yes | | Total | |
| base of hand |  | 1 |  | 1 |  | 2 |  |
| fingertips |  | 26 |  | 11 |  | 37 |  |
| fists |  | 10 |  | 12 |  | 22 |  |
| knuckles |  | 20 |  | 9 |  | 29 |  |
| nd |  | 19 |  | 8 |  | 27 |  |
| Total |  | 76 |  | 41 |  | 117 |  |
|  | | | | | | | |

| Chi-Squared Tests | | | | | | | |
| --- | --- | --- | --- | --- | --- | --- | --- |
|  | | Value | | df | | p | |
| Χ² |  | 6.695 |  | 5 |  | 0.244 |  |
| N |  | 117 |  |  |  |  |  |
|  | | | | | | | |

| Contingency Tables | | | | | | | |
| --- | --- | --- | --- | --- | --- | --- | --- |
|  | | Pollen/grass/dust allergy | | | |  | |
| Photograph no. | | No | | Yes | | Total | |
| 1 |  | 6 |  | 5 |  | 11 |  |
| 2 |  | 11 |  | 3 |  | 14 |  |
| 3 |  | 7 |  | 2 |  | 9 |  |
| 4 |  | 5 |  | 2 |  | 7 |  |
| 5 |  | 5 |  | 5 |  | 10 |  |
| 6 |  | 6 |  | 3 |  | 9 |  |
| 7 |  | 2 |  | 4 |  | 6 |  |
| 8 |  | 15 |  | 11 |  | 26 |  |
| Total |  | 57 |  | 35 |  | 92 |  |
|  | | | | | | | |

| Chi-Squared Tests | | | | | | | |
| --- | --- | --- | --- | --- | --- | --- | --- |
|  | | Value | | df | | p | |
| Χ² |  | 6.096 |  | 7 |  | 0.529 |  |
| N |  | 92 |  |  |  |  |  |
|  | | | | | | | |

|  | | Test | | Statistic | | df | | p-value | |
| --- | --- | --- | --- | --- | --- | --- | --- | --- | --- |
| using computer (hour per day at work) |  | Mann-Whitney |  | 1270.500 |  |  |  | 0.913 |  |
| using computer (hour per day after work) |  | Mann-Whitney |  | 942.500 |  |  |  | **0.026** |  |
|  | | | | | | | | | |
| Note.  For all tests, the alternative hypothesis specifies that group 1_4 is less than group 5_8. | | | | | | | | | |

1. Results of the relation between the manner of eye rubbing (indicated in the response to presented photographs 1-4 or 5-8) and time spent using a computer, tested individuals were not divided into patients and controls
