# Supplementary material for: Non-allergic eye rubbing is a major behavioral risk factor for keratoconus
Source: PLoS One. 2023 Apr 13;18(4):e0284454. doi: 10.1371/journal.pone.0284454 (PMC10101517; doi:10.1371/journal.pone.0284454)
Supplement: S7 Table — (DOCX) [file pone.0284454.s009.docx]

**S7 Table. Results of pathway enrichment analysis for genes whose expression in corneal epithelium was found to be related to the allergy status.** Note that these genes didn’t contribute to IgE-related pathways, such as ‘allergen dependent IgE bound FCERI aggregation’. No allergy-related inflammation of any kind has occurred (e.g., ‘interleukin-4 and interleukin-13 signaling’).

| Pathway | Fold Enrichment | Enrichment FDR | nGenes | Pathway Genes | Genes |
| --- | --- | --- | --- | --- | --- |
| Loss of Function of TGFBR1 in Cancer | 73.1814 | 0.0188 | 2 | 7 | TGFBR1, SMAD3 |
| Loss of Function of SMAD2/3 in Cancer | 73.1814 | 0.0188 | 2 | 7 | TGFBR1, SMAD3 |
| SMAD2/3 Phosphorylation Motif Mutants in Cancer | 85.3783 | 0.0188 | 2 | 6 | TGFBR1, SMAD3 |
| TGFBR1 KD Mutants in Cancer | 85.3783 | 0.0188 | 2 | 6 | TGFBR1, SMAD3 |
| Signaling by TGF-beta Receptor Complex in Cancer | 64.0337 | 0.0200 | 2 | 8 | TGFBR1, SMAD3 |
