# Supplementary material for: Non-allergic eye rubbing is a major behavioral risk factor for keratoconus
Source: PLoS One. 2023 Apr 13;18(4):e0284454. doi: 10.1371/journal.pone.0284454 (PMC10101517; doi:10.1371/journal.pone.0284454)
Supplement: Attachment — Submitted filename: Response to Reviewers.docx [file pone.0284454.s011.docx]

Poznan, February 14^th^, 2023

Georgios Labiris, MD, PhD
Academic Editor
PLOS ONE

Dear Editor,

Thank you for providing the constructive and helpful comments from the expert Reviewers for our manuscript ‘Non-allergic eye rubbing is a major behavioral risk factor for keratoconus’ (Ms. Ref. No.: PONE-D-22-34939). We appreciate that the Reviewers consider this work of interest to the readership of *PLOS ONE.*

We kindly ask for an amendment in ‘Funding Information’ as follows: ‘Supported by National Science Centre in Poland (<https://www.ncn.gov.pl/en>*),* Grants 2018/31/B/NZ5/03280 (to MG) and 2021/41/B/NZ5/02245 (to MG), and Polish Ministry of Science and Higher Education, Grant 6817/IA/SP/2018 (to RP and MR; Genomics Core Facility CeNT UW).’

Additionally, we ask for an amendment in the ‘Funders Statement’ as follows: ‘The funders had no role in study design, data collection and analysis, decision to publish, or preparation of the manuscript.’

Moreover, we kindly ask for an amendment in the ‘Data Availability’ statement as follows: ‘All proceeded clinical/environmental/behavioral/socioeconomic/molecular data, as minimal data set underlying the results described in our manuscript, are shared in a public repository Zenodo (DOI:10.5281/zenodo.7635600; https://doi.org/10.5281/zenodo.7635600).’

The revised manuscript with visible changes and point-by-point response to the Reviewers’ criticisms is enclosed for your consideration.

We greatly appreciate your assistance.

Sincerely,

Marzena Gajecka

Marzena Gajecka, Ph.D.

Professor

Institute of Human Genetics

Polish Academy of Sciences

Strzeszynska 32

60-479 Poznan, Poland

phone +48 61 6418310

mobile +48 603107587

email gamar@man.poznan.pl

*Journal Requirements:*

*When submitting your revision, we need you to address these additional requirements.*

*1. Please ensure that your manuscript meets PLOS ONE's style requirements, including those for file naming. The PLOS ONE style templates can be found at*

[*https://journals.plos.org/plosone/s/file?id=wjVg/PLOSOne_formatting_sample_main_body.pdf*](https://journals.plos.org/plosone/s/file?id=wjVg/PLOSOne_formatting_sample_main_body.pdf) *and* 
[*https://journals.plos.org/plosone/s/file?id=ba62/PLOSOne_formatting_sample_title_authors_affiliations.pdf*](https://journals.plos.org/plosone/s/file?id=ba62/PLOSOne_formatting_sample_title_authors_affiliations.pdf)

Completed as requested.

*2. We note that the grant information you provided in the ‘Funding Information’ and ‘Financial Disclosure’ sections do not match.*

*When you resubmit, please ensure that you provide the correct grant numbers for the awards you received for your study in the ‘Funding Information’ section.*

Completed as requested, the final version of Funding Information and Financial Disclosure is as follows:

‘Supported by National Science Centre in Poland (<https://www.ncn.gov.pl/en>), Grants 2018/31/B/NZ5/03280 (to MG) and 2021/41/B/NZ5/02245 (to MG), and Polish Ministry of Science and Higher Education, Grant 6817/IA/SP/2018 (to RP and MR; Genomics Core Facility CeNT UW).’

*3. Thank you for stating the following financial disclosure:*

*"Supported by National Science Centre in Poland (*[*https://www.ncn.gov.pl/en*](https://www.ncn.gov.pl/en)*), Grants 2018/31/B/NZ5/03280 (to MG) and 2021/41/B/NZ5/02245 (to MG)."*

*Please state what role the funders took in the study.  If the funders had no role, please state: "The funders had no role in study design, data collection and analysis, decision to publish, or preparation of the manuscript." If this statement is not correct you must amend it as needed.*

*Please include this amended Role of Funder statement in your cover letter; we will change the online submission form on your behalf.*

Completed as requested, the final version of Role of Funders statement is as follows:

‘The funders had no role in study design, data collection and analysis, decision to publish, or preparation of the manuscript.’

*4. Thank you for stating the following in the Acknowledgments Section of your manuscript:*

*"Next-generation sequencing was performed thanks to Genomics Core Facility CeNT UW using NovaSeq 6000 platform financed by the Polish Ministry of Science and Higher Education (decision no. 6817/IA/SP/2018 of 2018-04-10)."*

*We note that you have provided funding information that is not currently declared in your Funding Statement. However, funding information should not appear in the Acknowledgments section or other areas of your manuscript. We will only publish funding information present in the Funding Statement section of the online submission form.*

*Please remove any funding-related text from the manuscript and let us know how you would like to update your Funding Statement. Currently, your Funding Statement reads as follows:*

*"Supported by National Science Centre in Poland (*[*https://www.ncn.gov.pl/en*](https://www.ncn.gov.pl/en)*), Grants 2018/31/B/NZ5/03280 (to MG) and 2021/41/B/NZ5/02245 (to MG)."*

*Please include your amended statements within your cover letter; we will change the online submission form on your behalf.*

Completed as requested.

*5. In your Data Availability statement, you have not specified where the minimal data set underlying the results described in your manuscript can be found. PLOS defines a study's minimal data set as the underlying data used to reach the conclusions drawn in the manuscript and any additional data required to replicate the reported study findings in their entirety. All PLOS journals require that the minimal data set be made fully available. For more information about our data policy, please see* [*http://journals.plos.org/plosone/s/data-availability*](http://journals.plos.org/plosone/s/data-availability)*.*

*Upon re-submitting your revised manuscript, please upload your study’s minimal underlying data set as either Supporting Information files or to a stable, public repository and include the relevant URLs, DOIs, or accession numbers within your revised cover letter. For a list of acceptable repositories, please see* [*http://journals.plos.org/plosone/s/data-availability#loc-recommended-repositories*](http://journals.plos.org/plosone/s/data-availability#loc-recommended-repositories)*. Any potentially identifying patient information must be fully anonymized.*

*Important: If there are ethical or legal restrictions to sharing your data publicly, please explain these restrictions in detail. Please see our guidelines for more information on what we consider unacceptable restrictions to publicly sharing data:* [*http://journals.plos.org/plosone/s/data-availability#loc-unacceptable-data-access-restrictions*](http://journals.plos.org/plosone/s/data-availability#loc-unacceptable-data-access-restrictions)*. Note that it is not acceptable for the authors to be the sole named individuals responsible for ensuring data access.*

*We will update your Data Availability statement to reflect the information you provide in your cover letter.*

Completed as requested. We uploaded and already publish our study’s minimal underlying data set in a public repository accepted by PLOS ONE. Any potentially identifying patient information in this dataset is fully anonymized.

The revised Data Availability statement is as follows:

‘All proceeded clinical/environmental/behavioral/socioeconomic/molecular data, as minimal data set underlying the results described in our manuscript, are shared in a public repository Zenodo (DOI: 10.5281/zenodo.7635600; https://doi.org/10.5281/zenodo.7635600).’

*6. We note that you have stated that you will provide repository information for your data at acceptance. Should your manuscript be accepted for publication, we will hold it until you provide the relevant accession numbers or DOIs necessary to access your data. If you wish to make changes to your Data Availability statement, please describe these changes in your cover letter and we will update your Data Availability statement to reflect the information you provide.*

Completed as requested. We uploaded and already publish our study’s minimal underlying data set in public repository accepted by PLOS ONE. Any potentially identifying patient information in this dataset is fully anonymized.

Revised Data Availability statement is as follows:

‘All proceeded clinical/environmental/behavioral/socioeconomic/molecular data, as minimal data set underlying the results described in our manuscript, are shared in a public repository Zenodo (DOI: 10.5281/zenodo.7635600; https://doi.org/10.5281/zenodo.7635600).’

*7. We note that Figure 1 includes an image of a participant in the study.*

*As per the PLOS ONE policy (*[*http://journals.plos.org/plosone/s/submission-guidelines#loc-human-subjects-research*](http://journals.plos.org/plosone/s/submission-guidelines#loc-human-subjects-research)*) on papers that include identifying, or potentially identifying, information, the individual(s) or parent(s)/guardian(s) must be informed of the terms of the PLOS open-access (CC-BY) license and provide specific permission for publication of these details under the terms of this license. Please download the Consent Form for Publication in a PLOS Journal (*[*http://journals.plos.org/plosone/s/file?id=8ce6/plos-consent-form-english.pdf*](http://journals.plos.org/plosone/s/file?id=8ce6/plos-consent-form-english.pdf)*). The signed consent form should not be submitted with the manuscript, but should be securely filed in the individual's case notes.*

*Please amend the methods section and ethics statement of the manuscript to explicitly state that the patient/participant has provided consent for publication: “The individual in this manuscript has given written informed consent (as outlined in PLOS consent form) to publish these case details”.*

*If you are unable to obtain consent from the subject of the photograph, you will need to remove the figure and any other textual identifying information or case descriptions for this individual.*

Completed as requested. The signed Consent Form for Publication in a PLOS Journal is securely filed in our individual's case notes. The methods section and ethics statement of the manuscript were revised as follows:

‘The individual whose photographs are presented in Fig 1 in this manuscript has given written informed consent (as outlined in PLOS consent form) to publish the case details.’

*8. Please upload a new copy of Figure 3 to 4 and Supporting Figure S1 as the detail is not clear. Please follow the link for more information:*

[*https://blogs.plos.org/plos/2019/06/looking-good-tips-for-creating-your-plos-figures-graphics/*](https://blogs.plos.org/plos/2019/06/looking-good-tips-for-creating-your-plos-figures-graphics/)
[*https://blogs.plos.org/plos/2019/06/looking-good-tips-for-creating-your-plos-figures-graphics/*](https://blogs.plos.org/plos/2019/06/looking-good-tips-for-creating-your-plos-figures-graphics/)

Completed as requested. The figure files were uploaded to the Preflight Analysis and Conversion Engine (PACE) digital diagnostic tool, to ensure that PLOS requirements are met.

***Additional Editor Comments:***

*Both reviewers have indicated that the paper has potential after some corrections. Please read their comments below and address their suggestions.


[Note: HTML markup is below. Please do not edit.]

Reviewers' comments:

Reviewer's Responses to Questions*

***Comments to the Author*** *1. Is the manuscript technically sound, and do the data support the conclusions?

The manuscript must describe a technically sound piece of scientific research with data that supports the conclusions. Experiments must have been conducted rigorously, with appropriate controls, replication, and sample sizes. The conclusions must be drawn appropriately based on the data presented.*

*Reviewer #1: Yes*

*Reviewer #2: Yes*

*2. Has the statistical analysis been performed appropriately and rigorously?*

*Reviewer #1: Yes*

*Reviewer #2: Yes*

*3. Have the authors made all data underlying the findings in their manuscript fully available?

The* [*PLOS Data policy*](http://www.plosone.org/static/policies.action#sharing) *requires authors to make all data underlying the findings described in their manuscript fully available without restriction, with rare exception (please refer to the Data Availability Statement in the manuscript PDF file). The data should be provided as part of the manuscript or its supporting information, or deposited to a public repository. For example, in addition to summary statistics, the data points behind means, medians and variance measures should be available. If there are restrictions on publicly sharing data—e.g. participant privacy or use of data from a third party—those must be specified.*

*Reviewer #1: Yes*

*Reviewer #2: No*

We revised that aspect to make our data fully available. As the all proceeded data, constituting our study’s minimal underlying data set is extensive, we uploaded and already published the data in a public repository accepted by PLOS ONE. Any potentially identifying patient information in this dataset is fully anonymized.

The revised Data Availability statement is as follows:

‘All proceeded clinical/environmental/behavioral/socioeconomic/molecular data, as minimal data set underlying the results described in our manuscript, are shared in a public repository Zenodo (DOI: 10.5281/zenodo.7635600; https://doi.org/10.5281/zenodo.7635600).’

*4. Is the manuscript presented in an intelligible fashion and written in standard English?

PLOS ONE does not copyedit accepted manuscripts, so the language in submitted articles must be clear, correct, and unambiguous. Any typographical or grammatical errors should be corrected at revision, so please note any specific errors here.*

*Reviewer #1: Yes*

*Reviewer #2: Yes*

*5. Review Comments to the Author

Please use the space provided to explain your answers to the questions above. You may also include additional comments for the author, including concerns about dual publication, research ethics, or publication ethics. (Please upload your review as an attachment if it exceeds 20,000 characters)*

*Reviewer #1: I read with great interest the manuscript title: Non-allergic eye rubbing is a major behavioral risk factor for keratoconus, here authors, confirm what other studies have already shown, that rubbing is a very important cause in the etiopathogenesis of KC.
The authors demonstrate that Eye-rubbing causes CE damage and triggers cellular stress which through its influence on cell apoptosis, migration, and adhesion affects the KTCN phenotype.
Moreover, as in others studies male seems to be more frequents in KC population
I would like to congratulate authors for their work since I really believe Ophthalmologist should stil work hard to inform KC patients of their pathology and risk factors. (Baenninger et al. Do patients with keratoconus have minimal diseade knowledge. Cornea)
Some comments:*

*-I am not sure that conductive keratoplasty is an therapeutic option nowadays*

We thank the Reviewer for this valuable comment. We change the original sentence as follows:

“Penetrating keratoplasty (PK), deep anterior lamellar keratoplasty (DALK), and Bowman layer transplantation (BL transplantation) are treatment options in advanced stages of the disease[5]”.

*-Describe acronyms only once in the manuscript*

Completed as requested.

*-Please add in results a comparation between male vs female regarding stage of disease and compare in discussion with other papers in the literature*

Completed as requested. The comparison between male and female patients with KTCN regarding stage of disease, including clinical parameters such as K1, K2, Kmax, anterior and posterior elevations, and TCT are presented in the S1 Table. No statistical differences between genders were identified.

Results of comparison between male and female patients with KTCN are attached below.

|  | **Males with KTCN (n=97)** | | **Females with KTCN (n=21)** | |
| --- | --- | --- | --- | --- |
|  | **x** $\boldsymbol{\pm}$ **SD** | **Median** | **x**$\boldsymbol{\pm}$ **SD** | **Median** |
| UDVA OD | 0.49 ± 0.38 | 0.40 | 0.32 ± 0.28 | 0.20 |
| BDVA OD | 0.81 ± 0.35 | 0.90 | 0.73 ± 0.34 | 0.85 |
| UDVA OS | 0.43 ± 0.34 | 0.30 | 0.52 ± 0.37 | 0.40 |
| BDVA OS | 0.77 ± 0.35 | 0.90 | 0.81 ± 0.28 | 0.90 |
| K1 OD [D] | 45.18 ± 4.87 | 43.90 | 46.13 ± 6.31 | 44.0 |
| K2 OD [D] | 47.85 ± 5.99 | 46.10 | 49.07 ± 6.73 | 47.20 |
| Kmax OD [D] | 53.68 ± 9.32 | 51.30 | 55.02 ± 10.61 | 51.40 |
| anterior elevation OD [μm] | 23.14 ± 18.92 | 17.00 | 25.43 ± 16.27 | 24.00 |
| posterior elevation OD [μm] | 49.17 ± 36.42 | 39.00 | 58.05 ± 44.50 | 61.00 |
| TCT OD [µm] | 466.39 ± 54.28 | 473.50 | 460.62 ± 64.50 | 485.00 |
| thinnest epithelial thickness OD [μm] | 43.09 ± 5.45 | 44.00 | 43.50 ± 5.55 | 44.00 |
| K1 OS [D] | 45.54 ± 4.94 | 43.65 | 44.96 ± 3.43 | 44.40 |
| K2 OS [D] | 48.41 ± 5.91 | 46.45 | 47.84 ± 4.16 | 46.50 |
| Kmax OS [D] | 54.76 ± 9.49 | 52.15 | 53.51 ± 7.56 | 51.50 |
| anterior elevation OS [μm] | 26.44 ± 19.02 | 23.50 | 24.55 ± 15.42 | 20.50 |
| posterior elevation OS [μm] | 52.82 ± 33.64 | 50.00 | 52.25 ± 27.45 | 46.50 |
| TCT OS [µm] | 469.76 ± 54.66 | 473.50 | 474.80 ± 49.10 | 485.00 |
| thinnest epithelial thickness OS [μm] | 43.28 ± 5.44 | 44.00 | 44.57 ± 6.68 | 46.00 |
| AL OD [mm] | 24.03 ± 0.88 | 24.00 | 23.86 ± 0.91 | 23.60 |
| AL OS [mm] | 23.96 ± 0.85 | 23.90 | 23.76 ± 1.03 | 23.55 |
| IOP OD [mmHg] | 13.26 ± 3.16 | 13.00 | 12.72 ± 3.30 | 12.00 |
| IOP OS [mmHg] | 13.16 ± 3.30 | 13.00 | 12.94 ± 3.35 | 12.50 |

Abbreviations and symbols in the Table: x – average, SD – standard deviation, OD – oculus dexter, OS - oculus sinister, UDVA – uncorrected distance visual acuity, BDVA – best-corrected distance visual acuity, K1 – flat keratometric readings, K2 – steep keratometric readings, Kmax – maximum simulated keratometry, TCT – thinnest corneal thickness, AL - Axial length, IOP – intraocular pressure

We additionally added the following sentences to the Results in the revised manuscript:

‘No statistical differences in clinical parameters such as K1, K2, Kmax, anterior and posterior elevations, and TCT, have been found in comparison between male and female patients with KTCN, and between adult and adolescent patients with KTCN (S1 and S2 Tables).’

We also performed additional patients’ subgroup comparison analyses (males vs. females, adults vs. adolescents), and the findings are presented in S1, S2, S4 and S5 Tables, and we added the following statements:

‘No statistical differences in clinical parameters such as K1, K2, Kmax, anterior and posterior elevations, and TCT, have been found in comparison between male and female patients with KTCN, and between adult and adolescent patients with KTCN (S1 and S2 Tables).’

‘All analysed data are compiled in S3 Table, while additional results of comparison of male and female patients with KTCN and adult and adolescent patients with KTCN in aspect of selected behavioral, environmental, and socioeconomic factors are presented in S4 and S5 Tables, respectively.’

‘While we did not found differences in the manner of eye rubbing between adult and adolescent patients with KTCN, there was a significant difference considering sex of patients, that is male patients more frequently used knuckles, a base of hand, all fingers and/or a fist (as presented in photographs no. 5-8) (S4 and S5 Tables).’

‘Regarding the working environment, despite the lack of difference in the type of professional occupation between studied groups, we found that the presence of dust in the working environment was more frequently indicated by patients with KTCN (Table 2), and especially male patients with KTCN (S4 Table).’

*-Compare in more details your results with Moran et al study, especially and with the metaanalysis of Hashemi et al. Cornea 2020*

On our list of references the work of Moran et al. 2020 is numbered as [30]. This reference is already brought to discussion considering the following risk factors: eye rubbing, allergy and sleep position:

‘Previously the strong relation between eye rubbing and KTCN was reported in the case reports[32], case series[33], multivariate analyses[30], and a meta-analysis[9]. Here we verified this aspect and further characterized the eye rubbing behavior practiced by patients with KTCN.’

‘Here, the allergy has not been disclosed as a risk factor for KTCN. Regarding the aspect of allergy and atopy in patients with KTCN, it has been previously discussed as more prevalent in KTCN than in general population[30,44,45].’

‘Other, previously discussed factors as sleeping position and creating additional pressure on the eyeball[30] or smoking[31] weren’t confirmed in our study.’

The meta-analysis of Hashemi et al. 2020 is numbered on our reference list as [9].

We revised the statement in the Discussion as follows:

‘Previously the strong relation between eye rubbing and KTCN was reported in the case reports[32], case series[33], multivariate analyses[30], and a meta-analysis[9].

*- I would highlight the importance of early diagnosis, especially in children due to the risk of presenting in moderate-advanced stages of diagnosis and risk of progression*

We thank the Reviewer for this valuable comment. To address aspect of importance of early diagnosis we revised the following sentences in the Discussion:

‘We did not identify clinical features corelated with gender or age, however, the gender and age could potentially influence study results regarding the life habits.’

and we added the statement:

‘The absence of differences in clinical parameters between adult and adolescent patients indicates a rapid progression in the latter, pointing to the importance of increasing patient awareness as well as early diagnosis due to substantial risk of progression.’

*References:
1-Rocha de Lossada et al. Acta Ophthalmologica 2020
2-Ertan A & Muftuoglu O. Keratoconus clinical findings according to different age and gender groups. Cornea 2008
3-Leoni-MesplieS, et al. Scalability and severity of keratoconus in children. Am J Ophthalmol 2012
4- Fink BA et al. Differences in Keratoconus as a Function of Gender. Am J Ophthalmol 2005
5-Tharini B, et al. Keratoconus in pre-teen children: Demographics and clinical profile. Indian J Ophthalmol. 2022*

We thank the Reviewer for pointing to the publications, which have strengthened the quality of our discussion.

We added additional sentences to the discussion:

‘We did not identify clinical features corelated with sex or age, however, the sex and age could potentially influence study results regarding the life habits. The absence of differences in clinical parameters between adult and adolescent patients indicates a rapid progression in the latter[57–59], pointing to the importance of increasing patient awareness[60] as well as early diagnosis due to substantial risk of progression.’

‘Moreover, the absence of differences in clinical parameters between adult and adolescent patients, and male and female patients, in contrast to previous reports[38,61], enabled a superior appreciation of both, behavioral and molecular findings.’

**Other incorporated changes to the manuscript include: 1) the order and number of the references (seven additional references); 2) the number and order of the supplementary materials in the Supporting Information (four additional tables); 3) the number and order of figures (one additional figure cut out from the original Table 5); and 4) minor grammatical changes, as highlighted in the revised version.**

*Reviewer #2: The manuscript is well written and has some novel findings.
Presence of KC in lower educated population is due to selection bias. Advanced KC prevents people from higher education.*

We thank the Reviewer for this valuable comment. The following sentence was added to the Discussion:

‘In this paper, we presented the level of higher education as the only variable reducing the risk of KTCN (OR=0.52, univariate analysis), but this result should be treated with caution as it may be a consequence of selection bias.’

*Abstract: "we characterized them as features determining KTCN phenotype, and in particular the corneal epithelium" [lease change the sentence to be more clear especially about epithelium.*

Completed as requested.

The original text:

“Since the environmental, behavioral, and socioeconomic factors in the etiology of keratoconus (KTCN) remain poorly understood, we characterized them as features determining KTCN phenotype, and in particular the corneal epithelium (CE).”

We change to:

“Since the environmental, behavioral, and socioeconomic factors in the etiology of keratoconus (KTCN) remain poorly understood, we characterized them as features influencing KTCN phenotype, and especially affecting the corneal epithelium (CE).”

We also performed additional patients’ subgroup comparison analyses (males vs. females, adults vs. adolescents), and the findings are presented in S1, S2, S4 and S5 Tables, and we added the following statements:

‘No statistical differences in clinical parameters such as K1, K2, Kmax, anterior and posterior elevations, and TCT, have been found in comparison between male and female patients with KTCN, and between adult and adolescent patients with KTCN (S1 and S2 Tables).’

‘All analysed data are compiled in S3 Table, while additional results of comparison of male and female patients with KTCN and adult and adolescent patients with KTCN in aspect of selected behavioral, environmental, and socioeconomic factors are presented in S4 and S5 Tables, respectively.’

‘While we did not found differences in the manner of eye rubbing between adult and adolescent patients with KTCN, there was a significant difference considering sex of patients, that is male patients more frequently used knuckles, a base of hand, all fingers and/or a fist (as presented in photographs no. 5-8) (S4 and S5 Tables).’

‘Regarding the working environment, despite the lack of difference in the type of professional occupation between studied groups, we found that the presence of dust in the working environment was more frequently indicated by patients with KTCN (Table 2), and especially male patients with KTCN (S4 Table).’

**Other incorporated changes to the manuscript include: 1) the order and number of the references (seven additional references); 2) the number and order of the supplementary materials in the Supporting Information (four additional tables); 3) the number and order of figures (one additional figure cut out from the original Table 5); and 4) minor grammatical changes, as highlighted in the revised version.**

*6. PLOS authors have the option to publish the peer review history of their article (*[*what does this mean?*](https://journals.plos.org/plosone/s/editorial-and-peer-review-process#loc-peer-review-history)*). If published, this will include your full peer review and any attached files.


If you choose “no”, your identity will remain anonymous but your review may still be made public.*


***Do you want your identity to be public for this peer review?*** *For information about this choice, including consent withdrawal, please see our* [*Privacy Policy*](https://www.plos.org/privacy-policy)*.*

*Reviewer #1: No*

*Reviewer #2:* ***Yes:*** *Hesam Hashemian*

*[NOTE: If reviewer comments were submitted as an attachment file, they will be attached to this email and accessible via the submission site. Please log into your account, locate the manuscript record, and check for the action link "View Attachments". If this link does not appear, there are no attachment files.]*

*While revising your submission, please upload your figure files to the Preflight Analysis and Conversion Engine (PACE) digital diagnostic tool,* [*https://pacev2.apexcovantage.com/*](https://pacev2.apexcovantage.com/)*. PACE helps ensure that figures meet PLOS requirements. To use PACE, you must first register as a user. Registration is free. Then, login and navigate to the UPLOAD tab, where you will find detailed instructions on how to use the tool. If you encounter any issues or have any questions when using PACE, please email PLOS at* [*figures@plos.org*](mailto:figures@plos.org)*. Please note that Supporting Information files do not need this step.*

*In compliance with data protection regulations, you may request that we remove your personal registration details at any time.* [*(Remove my information/details)*](https://www.editorialmanager.com/pone/login.asp?a=r)*. Please contact the publication office if you have any questions.*
